# Enterohemorrhagic Escherichia coli O157 in North Africa region: a threat require advanced investigation

**DOI:** 10.11604/pamj.2014.19.26.4825

**Published:** 2014-09-12

**Authors:** Mohamed Ahmed, Jennifer Van Velkinburgh

**Affiliations:** 1 Department of Microbiology and Parasitology, Faculty of Veterinary Medicine, University of Tripoli, Tripoli, Libya; 2 Initiative for Collaboratory Biomedical Research, Santa Fe, New Mexico, USA

**Keywords:** Enterohemorrhagic, Escherichia coli, diarrheagenic

## To the editors of the Pan African Medical Journal

Several concerns exist regarding the prevalence, incidence, distribution and infectiousness of bacterial pathogen Enterohemorrhagic *E. coli* (EHEC) O157 in the North African region. EHEC O157 strains have been detected worldwide in animals as well as humans; the related infections have a complicated clinical course with occasionally fatal outcome, and the documented strains show remarkable variability between and within different countries [1–4]. Animals, cattle in particular, are a major reservoir and an important transmission vector of feacal *E. coli* O157 [5–7]. A limited amount of studies from Africa have reported diarrheagenic *E. coli* from human and non-human reservoirs but the true extent and burden *E. coli* 0157 is still unclear and possibly underestimated [5–8]. Below is a summary and comparison of recent published information on the status of *E. coli* 0157 in humans and animals in the North African region (Table 1).

**Table 1:** Summary of the prevalence and status of *E. coli* O157 in North African region

Country	Prevalence of O157 in		References
	Animals source & products	Humans	
**Libya**	**6.2%** (cattle faecal source)	**7.0%** (DC)	7
	**20.3%** (chicken meat)	**4.4%** (C)	*11*
		**0.7%** (DC)	12
		**0%** (DC)	13
**Egypt**	**2%** (raw milk)	**6.7%** (DC)	14
	**3.4%** (animals (beef, chicken and milk) product)	_	4
	**26.7%** (beef products)	_	15
	**1.42%** (faecal source)	_	
**Algeria**	**0.4%** (bovine meat)	_	16
	**7.8%** (bovine carcasses)	_	2
**Tunisia**	_	**10.4%** (DC)	17
		**11.1%** (C)	

C: children; DC: diarrhogenic children; No: available data

Since the first reported outbreaks in Africa in the 1990s very limited information has been published regarding *E. coli* O157 and related outbreaks in humans [5, 9]. In recent decades, few studies have investigated the prevalence of *E. coli* O157 in north African region, mostly focused on animals and food products (bovine-cattle and chicken) or/and from human stools (diarrheic and non diarrheic children) (Table 1). Most of North African studies have applied basic and recommended laboratory techniques with very limited application of PCR technology and absence of thorough epidemiological risk factors analysis [10]. The variation between different countries could be related to different risk factors which might have contributed to such variation even within countries and for both humans and animals in North Africa region. (Table 1]. Cattle, especially under stress conditions, are a major reservoir of feacal *E. coli* O157, and an important environmental factor contributing to the zoonotic transmission of this bacterial pathogen in Africa by direct contact with feacal matter from shedding infected cattle or indirectly through ingestion of contaminated food products with faecal matters [5, 7, 11]. The North African countries share environmental, many economic and social features, which may have led to the close and lack documented status of *E. coli* O157 of some of these studies [12–17].

Most of data related to public health concerns from North Africa, are largely based on standard laboratory applications. This approach, with the exception of some researchers’ efforts has resulted in most publications neglecting the key importance of applying the data from epidemiological studies to identifying and resolving infections and outbreak [7]. PCR-based technology has become the gold standard for laboratory, diagnostics and epidemiological purposes across the globe; however, the standard and basic laboratory methods that have documented reliability are still recommended for use, especially in developing countries with insufficient accessibility to and expertise in the modern molecular technologies [2, 9, 10]. This letter has also brought forth a very important issue that also needs to be discussed: the traditional (and unfortunately regressive) mindset held by a portion of the scientific and medical community in some developing countries, especially developing countries, that is encumbering the overall efforts to advance these nations’ scientific and medical programs on a global scale. In order for the researchers of these countries to make meaningful contributions on a global scale, they must learn and employ the newest technologies and networking ability to the benefit of their national health and laboratory quality [18].

The current status of EHEC O157 in North African region is unclear and the public health risk concern remains uninvestigated. There is need to implement epidemiological researches and monitoring studies to determine the zoonotic risk of this pathogen from different animals, mainly in cattle. Such important investigations and interventions will provide health authorities with excellent data and important epidemiological information related to this pathogen. Developing countries should promote science and public health by setting up a sustainable future cadre of advanced thinking researchers who are willing and capable of embracing new technologies for the betterment of their societies.
